# Hospitalization rates and outcome of invasive bacterial vaccine-preventable diseases in Tuscany: a historical cohort study of the 2000–2016 period

**DOI:** 10.1186/s12879-018-3316-1

**Published:** 2018-08-13

**Authors:** Elena Chiappini, Federica Inturrisi, Elisa Orlandini, Maurizio de Martino, Chiara de Waure

**Affiliations:** 1 Anna Meyer Children’s University Hospital, Department of Health Sciences, University of Florence, Florence, Italy; 2 Department of Epidemiology & Biostatistics, VU University Medical Center (VUmc), Amsterdam, the Netherlands; 3 Tuscany Regional Government Department of Right to Health and Solidarity Policies, Information Technology Section, Florence, Italy; 4 Department of Experimental Medicine, University of Perugia, Piazzale Gambuli 1, 06132 Perugia, Italy

**Keywords:** Invasive bacterial diseases, Hospitalization, trend

## Abstract

**Background:**

Invasive bacterial diseases (IBD) are a serious cause of hospitalization, sequelae and mortality. Albeit a low incidence, an increase in cases due to *H. influenzae* was registered in the past 4 years and, in the Tuscany region, an excess of cases due to *N. meningitidis* since 2015 is alarming. The purpose of this study is to deepen the knowledge of IBD epidemiology in Tuscany with particular attention to temporal trends.

**Methods:**

Tuscan residents hospitalized for IBD from January 1st 2000 to March 18th 2016 were selected from the regional hospital discharge database based on ICD-9-CM codes. Age-specific and standardized hospitalization rates were calculated together with case-fatality rates (CFRs). A time-trend analysis was performed; whereas, prognostic factors of death were investigated through univariable and multivariable analyses.

**Results:**

The average standardized hospitalization rates for invasive meningococcal diseases (IMD), invasive pneumococcal diseases and invasive diseases due to *H. influenzae* from 2000 to 2016 were 0.6, 1.8, and 0.2 per 100,000, respectively. The average CFRs were 10.5%, 14.5% and 11.5% respectively with higher values in the elderly. Older age was significantly associated with higher risk of death from all IBD. A significant reduction in hospitalization rates for IMD was observed after meningococcal C conjugate vaccine introduction. The Annual Percentage Change (APC) was -13.5 (95% confidence interval (CI) -22.3; -3.5) in 2005–2013 but has risen since that period. Furthermore, a significant increasing trend of invasive diseases due to *H. influenzae* was observed from 2005 onwards in children 1–4 years old (APC 13.3; 95% CI 0; 28.3).

**Conclusions:**

This study confirms changes in the epidemiology of invasive diseases due to *H. influenzae* and IMD. Furthermore, attention is called to the prevention of IBD in the elderly because of the age group’s significantly higher rate of hospitalizations and deaths for all types of IBD.

**Electronic supplementary material:**

The online version of this article (10.1186/s12879-018-3316-1) contains supplementary material, which is available to authorized users.

## Background

Invasive bacterial diseases (IBD) are an important public health issue and cause a serious burden in several countries, particularly among young persons and the elderly. The most common IBD clinical manifestations are septicemia and meningitis, with the first occurring even without the presence of the second, accounting together for 170,000 annual deaths worldwide [1, 2]. Meningitis is a severe infection of the meninges and can rapidly progress from the sudden onset of non-specific symptoms (including fever, nausea, vomiting, and neck stiffness) to death in 24 h. As many as 20–50% of the survivors may have permanent sequelae, such as hearing loss, amputation, or neurological and behavioral impairments [3–5]. Septicemia is a life-threatening condition that can cause tissue damage, organ failure, and death [6]. The three most common etiological agents of IBD are *Haemophilus influenzae*, *Streptococcus pneumoniae*, and *Neisseria meningitidis*. They are carried asymptomatically in the human nasopharynx and transmitted by aerosol droplets or secretions during close or lengthy contact. *H. influenzae* may be non-encapsulated (non-typeable) or encapsulated with a polysaccharide capsule. In the latter case, six serotypes (a-f) are recognized, with *H. influenzae* serotype b (Hib) being the most pathogenic. Before the availability of Hib conjugate vaccines in the late 1990s, *H. influenzae* was causing the majority of bacterial meningitis and IBD, while it now accounts for only 2–7% of cases [7–10]. Invasive diseases due to *H. influenzae* are most common in children below 5 years of age and rare in adolescents and adults [11]. With the decline of cases due to *H. influenzae*, *S. pneumoniae* became the leading cause of IBD, especially among children younger than 5 years of age, elderly and people with chronic diseases or immuno-compromised [12]. Out of 93 known *S. pneumoniae* serotypes, only 20–30 are responsible for the majority of invasive pneumococcal diseases (IPD) worldwide [13]. Furthermore, *S. pneumoniae* is known as the leading cause of community-acquired pneumonia, and the lethality associated with bacteriaemic pneumococcal pneumonia is 6–20% [14]. The 7-valent pneumococcal conjugate vaccine (PCV7) for infants and young children was licensed in Europe in 2001, leading to a reduction in hospitalization rates for all *S. pneumoniae* –related diseases in children [15–17]. With the replacement of PCV7 with the 13-valent pneumococcal conjugate vaccine (PCV13) in 2010–2011 and the extension of vaccination to elderly and people at risk [18], it is likely that *N. meningitidis* will become a major agent of IBD worldwide, in particular if vaccination coverage will not reach high levels. Moreover, *N. meningitidis* is the only bacterium able to produce epidemics of meningitis. Incidence rates of invasive meningococcal diseases (IMD) are generally highest in children below 5 years of age followed by adolescents and young adults. Thirteen serogroups of *N. meningitidis* can be identified on the basis of the polysaccharide capsule but only six are responsible for most IMD cases: A, B, C, W135, X, and Y [2]. The distribution of these serogroups varies geographically, likely because of differences in population immunity and environmental factors. In Europe, serogroup B (MenB) is the main cause of IMD, accounting for up to 80% of cases in some countries, followed by serogroups C (MenC) and Y (MenY) [2, 19]. With the introduction of the MenC conjugate vaccine (MCC) in the immunization schedules of several European countries, a significant decline in the incidence of MenC diseases has occurred over the past 10 years [19–21].

Nowadays, in Italy, PCV13 is given, together with Hib vaccine, in three doses within the first year of age (3, 5–6, 11–13 months of age) [22]; whereas MCC is delivered in a single dose at 13–15 months of age with a catch-up at 12–14 years of age [23]. In 2014–2015, a multicomponent MenB vaccine (4CMenB) has been introduced and is currently given in two, three or four doses depending on child’s age at the time of vaccination. Nevertheless, until the launch of the new National Immunization Plan in January 2017, MenB vaccine has been offered free of charge only in few Italian regions [23–25]. Incidence rates for IBD are relatively low in all Italian regions. In 2014, national incidence rates were as follows: 0.17 per 100,000 for invasive *H. influenzae* diseases, 0.27 per 100,000 for IMD, and 1.57 per 100,000 for IPD. However, an increase in the incidence rates of invasive *H. influenzae* diseases was registered from 2011 [26]. Moreover, in particular in the Tuscany region, an excess of IMD cases was registered from January 2015 onwards [27]. Health authorities responded to the increasing number of cases with an extraordinary vaccination campaign [28, 29].

The present study aims to study the epidemiology of IBD in Tuscany, with particular attention to temporal trends. For the three types of IBD, over a 16-year period, we estimated: i) age-specific and standardized hospitalization rates and their relationship with vaccination coverage; ii) age-specific case-fatality rates (CFR); and iii) time-trends of age-specific hospitalization rates. Additionally, prognostic factors for death were investigated.

## Methods

### Study design, case definition and data collection

This is a historical cohort study conducted in Tuscany, an Italian region with a population of 3.7 million people. The regional hospital discharge database was accessed in order to identify patients admitted with a diagnosis of IBD due to *N. meningitidis*, *S. pneumoniae* or *H. influenzae* from January 1st 2000 to March 18th 2016. The database relies on the International Classification of Disease, Ninth Revision, Clinical Modification (ICD-9-CM) system that is currently used in Italy [30]. The project has been approved by the Ethics Committee of the “Azienda Ospedaliero-Universitaria Meyer” of Florence on October 4th 2010 (authorization number 2010/7880).

Eligible patients were retrospectively searched in the regional hospital discharge database using the following ICD-9-CM codes for primary or secondary diagnosis:

- IMD: 036.0 (meningococcal meningitis), 036.1 (meningococcal encephalopathy), 036.2 (meningococcal septicemia), 036.40 (unspecified meningococcal carditis), 036.41 (meningococcal pericarditis), 036.42 (meningococcal endocarditis), 036.43 (meningococcal myocarditis), 036.81 (meningococcal optic neuritis), 036.82 (meningococcal arthropathy), 036.89 (other specified forms of meningococcal infection), and 036.9 (unspecified meningococcal infection);
- IPD: 038.2 (pneumococcal septicemia), and 320.1 (pneumococcal meningitis);
- invasive *H. influenzae* diseases: 038.41 (septicemia due to *H. influenzae*), and 320.0 (Haemophilus meningitis).

Hospital discharge records of eligible patients included the following data: details of the admitting hospital, age, gender, nationality, region of residence, date of admission, one primary and five secondary diagnoses, surgical and other procedures, date and type of discharge. All hospitalized patients living in Tuscany and discharged from a Tuscan hospital, with one of the ICD-9-CM codes described above in primary or secondary diagnosis, were included in the study. One day-hospitalizations were excluded. A cross-check of data included in the hospital discharge record of included patients was conducted to avoid duplicates due to patients being transferred from a hospital to another one. With respect to the population at risk, the number of people residing in Tuscany during the study period (at January 1st of each year), stratified by age and gender, was taken from the Italian National Statistical Institute (ISTAT) database [31]. Data on vaccination coverage of *H. influenzae* (Hib), *S. pneumoniae* (PCV) and *N. meningitidis* (MCC) at 24 months of age were searched on the Italian Ministry of Health database and ICONA studies [32–34].

### Statistical analysis

Continuous variables were summarized using mean ± standard deviation (SD), whereas categorical variables were reported as absolute and relative frequencies. Statistical analyses were carried out using STATA software version 13.1 except as otherwise specified.

#### Descriptive analysis

Age-specific hospitalization rates (HR), stratified for type of invasive disease, were calculated as cases per 100,000 together with 95% confidence intervals (95% CI). Resident population at January 1st of each year was used for the calculation. Standardized hospitalization rates (SHR) and their 95% CI were considered to compare hospitalizations between years. Standardization was performed with respect to age and gender using the Italian population (latest available data from 2016) as external weight. 95% CIs were obtained as standardized rate ± 1.96*standard error (SE), and SE was calculated with the following Armitage and Berry formula [35]:

$$ \sqrt{\frac{\sum \frac{\left({T}_ix{N}_i^2 xK\right)}{n_i}}{{\left(\sum {N}_i\right)}^2}} $$

T_i_ = crude rate for each age class

N_i_ = size of the reference population in each age class

n_i_ = size of the study population in each age class

K = multiplication factor (100,000).

Patients were stratified into seven age groups following the classification adopted by the National Surveillance System: <1, 1–4, 5–9, 10–14, 15–24, 25–64, ≥65 years of age. Case-fatality rates (CFR) were calculated dividing the number of deaths by the number of cases and were presented stratified for type of invasive disease.

#### Time-trend analysis

Changes in overall and age-specific HRs from 2000 to 2015 were assessed by joinpoint (JP) regression according to Kim’s method [36]. Data regarding 2016 were excluded from this analysis because they were partial. A joinpoint represents the time point when a significant trend change is detected. Time changes were expressed in terms of Annual Percent Change (APC) with 95% CI. The null hypothesis was tested using a maximum of three changes in the slope with an overall significance level of 0.05 divided by the number of joinpoints in the final model. Joinpoint Regression program version 4.3.1 was used to carry out the analysis.

#### Health outcome analysis

Chi-square test was used to assess the relationships between the health outcome (dead, alive) and independent variables such as: age (<5, 5–17, 18–64, ≥65 years of age), gender, nationality (Italian, non-Italian) and Charlson Index [37]. The Charlson Index was used as a proxy of comorbidity and calculated according to the algorithms developed by Quan et al. [38] looking at Enhanced ICD-9-CM Coding in primary and secondary diagnoses. The STATA additional software package “charlson” was used for this calculation. Variables with *p*-values below 0.25 at the univariable analysis were entered in a logistic regression model. The results were shown in terms of Odds Ratio (OR) and 95% CI. All the analyses and models were carried out stratifying by type of invasive disease.

## Results

### Descriptive analysis

A total of 1691 patients with IBD were hospitalized between January 1st 2000 and March 18th 2016 at 52 hospitals in Tuscany region; 107 were residents outside the region and excluded from further analysis. Among the residents, 288 were children and adolescents (<18 years), and 1296 were adults and elderly (≥18 years). Additional file 1: Table S1 summarizes the study population’s characteristics. Most children and adolescents were admitted to the Anna Meyer Children’s University Hospital in Florence (*n* = 127; 44.1%). More than half of the hospitalizations of children and adolescents were due to IMD (*n* = 153; 53.1%) followed by IPD (*n* = 112; 38.9%). Children’s and adolescents’ mean age was 4.8 ± 5.2 years and the mean hospitalization length was 16 ± 15.4 days; 56.9% of them were males and 92% were Italian. Among adults and elderly, IPD was the most common IBD (*n* = 1017; 78.5%). Their mean age was 61.3 ± 18 years, males and females were equally represented and the majority was Italian. The mean length of hospitalization was 12 ± 8.9 days. Invasive *H. influenzae* diseases accounted for approximately 8% of cases in both groups. Data on vaccination coverage at 24 months of age in Tuscany were available for Hib from 2003 to 2015. Hib average coverage during the study period was of 94% but two drops to 88% were registered in 2003 and 2010. On the contrary, data for PCV and MCC vaccination coverage were retrieved only for 2003, 2008 and 2013–2015. Vaccination coverage was very low in 2003 and 2008, whereas it was between 92.9 and 94% for PCV and between 87.8 and 90.9% for MCC in 2013–2015.

**Fig. 1:**
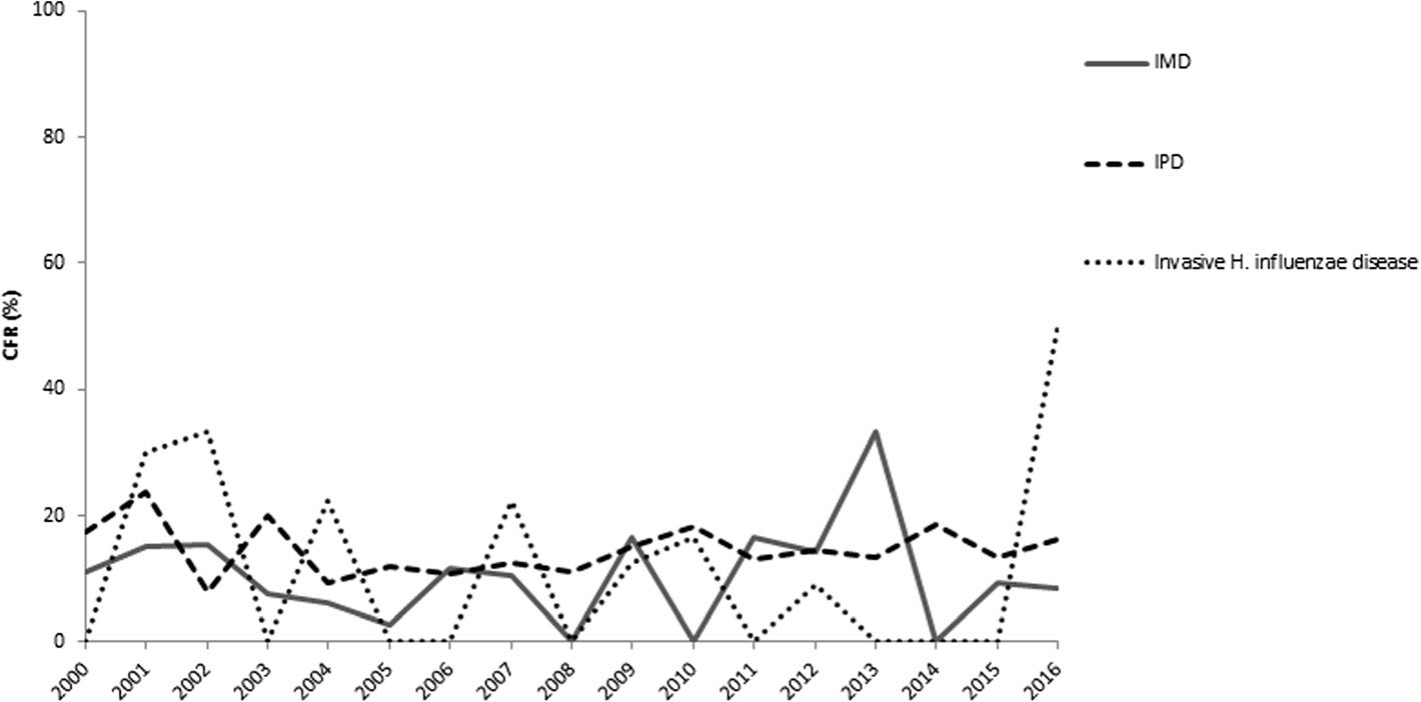
Case-fatality rates (CFRs) per type of IBD from 2000 to 2016

**Fig. 2:**
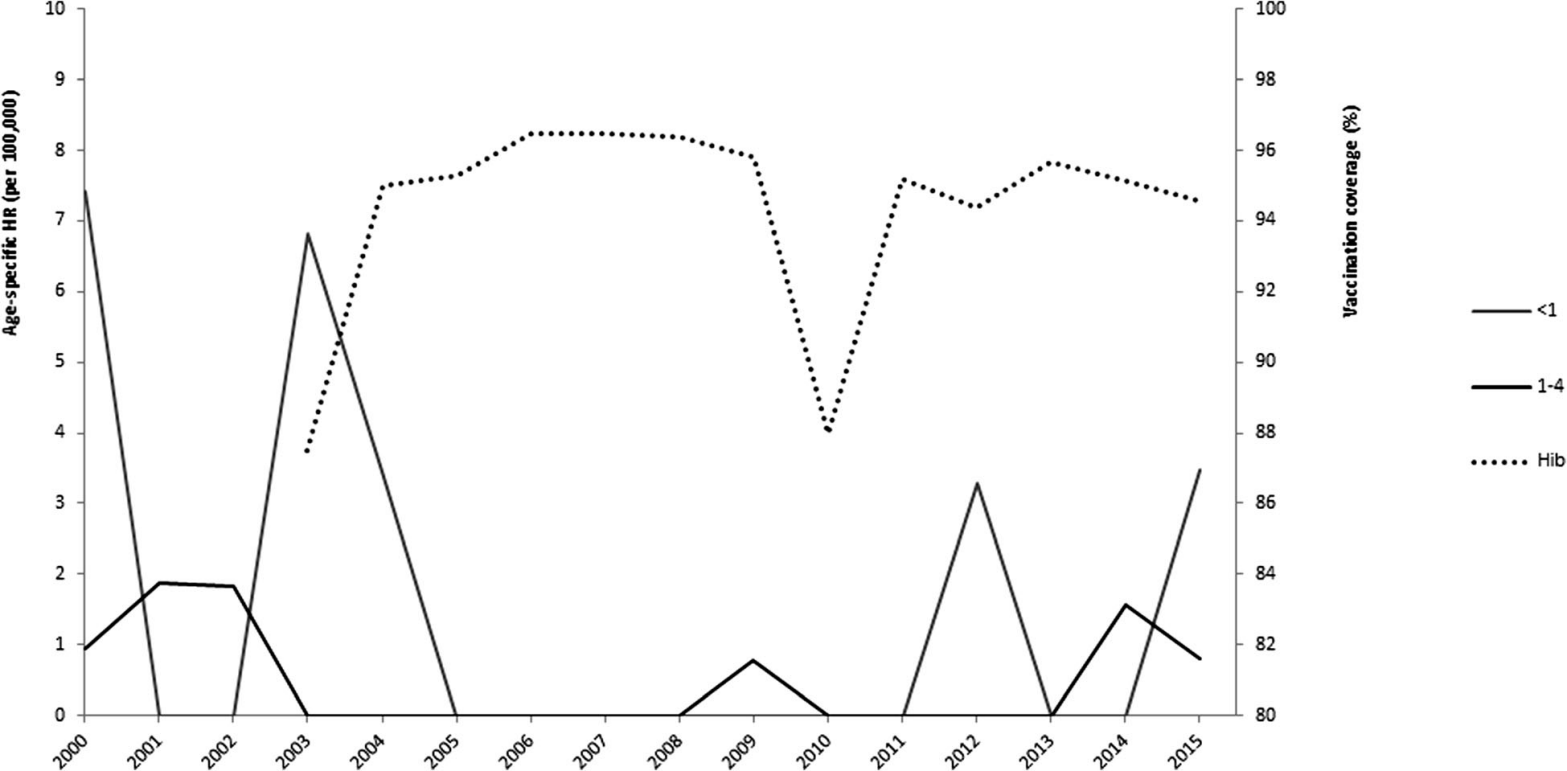
Age-specific HRs of invasive *H. influenzae* diseases in children below 4 years of age and Hib vaccination coverage in Tuscan children from 2003 to 2015

More than one-quarter (*n* = 86; 26.1%) of the 330 hospitalizations for IMD occurred in children less than 4 years of age. No child <1 year of age died, while five died in the 1–4 years age group, yielding a CFR of 8.2% in this age group. People from 15 to 24 and from 25 to 64 years of age accounted for almost half (*n* = 158; 47.9%) of IMD hospitalizations, with CFRs of 8.0% and 9.6% respectively. The highest number of deaths (11 deaths out of 42 cases) was registered in elderly (≥65 years of age) with a CFR of 26.2%. More than half (*n* = 581; 51.5%) of the 1129 hospitalizations for IPD occurred in people ≥65 years of age with more than three-quarters (*n* = 122; 75.3%) of all deaths due to this infection, yielding a CFR of 21%. Similarly, almost half (*n* = 61; 48.8%) of the 125 hospitalizations for invasive *H. influenzae* diseases occurred in the ≥65 years age group. All deaths for this IBD were registered in this age group (CFR: 21.3%) except one in the 1–4 years age group (CFR: 11.1%) (Table 1). During the whole study period, CFR due to IMD had the lowest mean value (10.5%, min 0.0%, max 33.3%). IPD and invasive *H. influenzae* diseases presented a mean CFR of 14.5% (min 7.8%, max 23.8%) and 11.5% (min 0.0%, max 50.0%) (Fig. 1). Table 2 reports SHRs for type of IBD and year. The mean SHR for IMD was 0.6 per 100,000, with two peaks around 1 per 100,000 in 2004–2005 and in 2015. IPD had a mean SHR of 1.8 per 100,000, ranging from 1.4 per 100,000 in 2010 to 2.2 per 100,000 in 2012. During the study period, invasive *H. influenzae* diseases had SHRs always below or equal to 0.3 per 100,000 (mean SHR: 0.2 per 100,000), with higher values in 2000–2005, 2012 and 2014 (Table 2). In the latter, the age-specific HRs for children <1 year of age and from 1 to 4 years of age showed peaks up to 7 per 100,000 children. The relationship between data on vaccination coverage and age-specific HRs for invasive *H. influenzae* diseases is shown in Fig. 2. On the contrary, the relationship between PCV and MCC vaccination coverage and HRs was not shown because of lack of data.

**Table 1:** Absolute and relative frequencies of cases and deaths, and CFRs by age group due to IBD in Tuscany from 2000 to 2016^a^ (*N* = 1584)

Age group	IMD			IPD			Invasive *H. influenzae* diseases		
	Cases n (%)	Deaths n (%)	CFR	Cases n (%)	Deaths n (%)	CFR	Cases n (%)	Deaths n (%)	CFR
<1 year	25 (7.6)	0 (0.0)	0.0	29 (2.6)	0 (0.0)	0.0	7 (5.6)	0 (0.0)	0.0
1–4 years	61 (18.5)	5 (16.1)	8.2	52 (4.6)	0 (0.0)	0.0	9 (7.2)	1 (7.1)	11.1
5–9 years	22 (6.7)	1 (3.2)	4.5	22 (2.0)	0 (0.0)	0.0	4 (3.2)	0 (0.0)	0.0
10–14 years	22 (6.7)	0 (0.0)	0.0	5 (0.4)	1 (0.6)	20.0	2 (1.6)	0 (0.0)	0.0
15–24 years	75 (22.7)	6 (19.4)	8.0	14 (1.2)	1 (0.6)	7.1	2 (1.6)	0 (0.0)	0.0
25–64 years	83 (25.1)	8 (25.8)	9.6	426 (37.7)	38 (23.5)	8.9	40 (32.0)	0 (0.0)	0.0
≥65 years	42 (12.7)	11 (35.5)	26.2	581 (51.5)	122 (75.3)	21.0	61 (48.8)	13 (92.9)	21.3
All ages	330 (100.0)	31 (100.0)	9.4	1129 (100.0)	162 (100.0)	14.3	125 (100.0)	14 (100.0)	11.2

^a^Data refer up to March 2016

*IMD* invasive meningococcal diseases, *IPD* invasive pneumococcal diseases, *CFR* case-fatality rate

**Table 2:** Summary table of HRs (per 100,000) standardized for age and gender

Years	IMD	IPD	Invasive *H. influenzae* diseases
2000	0.552	1.505	0.246
2001	0.593	1.789	0.307
2002	0.822	1.808	0.252
2003	0.373	2.057	0.329
2004	0.953	1.780	0.285
2005	1.067	2.046	0.217
2006	0.486	1.799	0.162
2007	0.557	1.920	0.251
2008	0.488	1.896	0.063
2009	0.508	1.748	0.202
2010	0.437	1.440	0.158
2011	0.352	2.002	0.084
2012	0.381	2.170	0.281
2013	0.250	1.741	0.109
2014	0.492	1.621	0.279
2015	0.904	2.048	0.205
2016^a^	0.336	0.783	0.051

^a^Data refer up to March 2016

*HR* hospitalization rate, *IMD* invasive meningococcal diseases, *IPD* invasive pneumococcal diseases

### Time-trend analysis

The joinpoint analysis by type of IBD and age group is shown in Tables 3, 4 and 5. As far as IMD is concerned, considering all ages, joinpoints were found in 2005 and 2013 showing a significant decreasing trend in 2005–2013 (APC -13.4; 95% CI -22.3; -3.5; *p* < 0.0001) and a positive but non-significant trend before and after this time. No joinpoints or significant trends were shown for children up to 9 years old. However, while infants <1 year of age had a positive APC (APC 3.1; 95% CI -5.1; 12.1), HRs for the 1–4 years and 5–9 years age groups tended to decrease (APC -5.9 and -7.6 respectively). The greatest reduction was found in the 10–14 years age group, whose APC decreased from 7.6 (95% CI -27.7; 60.2) in 2000–2004 to -8.5 (95% CI -15.9; -0.4; *p* < 0.0001) afterwards. No joinpoints were found in the other age groups. With respect to IPD, the overall trend was stable over time (APC 0.7; 95% CI -0.6; 2.1). Infants <1 year of age had an overall significant decreasing trend (APC -9.4; 95% CI -16; -2.3; *p* < 0.0001) (data not shown) with a joinpoint in 2004 and an APC of -16.7 in 2004–2015 (95% CI -28; -3.6; *p* < 0.0001). Children from 1 to 4 years of age also had a negative but non-significant APC (APC -7; 95% CI -13.7; 0.2). Joinpoints were found in the 10–14 years age group in 2002, 2009 and 2012, with positive and negative fluctuating trends. Young adults in the 15–24 years age group showed a joinpoint in 2003: APC changed from 95.2 (95% CI -50.5; 670.3) in 2000–2003 to -13.4 (95% CI -22.5; -3.3; *p* < 0.0001) afterwards. No joinpoints were found in adults and elderly (positive non-significant trends). For invasive *H. influenzae* diseases, there was an overall decreasing but non-significant trend (APC -1.5; 95% CI -5.3; 2.4). Joinpoints were found in all age groups except for adults and elderly. Infants <1 year of age had a significant decreasing trend in 2000–2008 (APC -17.5; 95% CI -30.5; -2.1; *p* < 0.0001) followed by an increasing but non-significant trend. Joinpoints were found in children from 1 to 4 years of age in 2002 and 2005: a significant increase was seen in 2005–2015 (APC 13.3; 95% CI 0.0; 28.3; *p* < 0.0001). On the contrary, the trend decreased for children 5–9 years old in 2008–2015 (APC -15.1; 95% CI -24; -5.1; *p* < 0.0001). In the 10–14 years age group, joinpoints were found in 2003, 2009 and 2012, with positive and negative fluctuating trends. Young adults in the 15–24 years age group had an overall significant decreasing trend (APC -3.4; 95% CI -5.5; -1.4; *p* < 0.0001) (data not shown) with a joinpoint in 2003 and an APC of -19.3 in 2003–2015 (95% CI -30.4; -6.5; *p* < 0.0001). An exemplary time-trend change is illustrated in Additional file 2: Figure S1.

**Table 3:** Findings of the joinpoint regression for IMD by age group

Age group	Joinpoint	Years Range	APC (95% CI)	*p*-value
All age groups	2005	2000–2005	9.9 (-5.3;27.5)	0.2
	2013	2005–2013	-13.4 (-22.3; -3.5)	< 0.0001
		2013–2015	80 (-7.3; 249.5)	0.1
<1 year	No JP	2000–2015	3.1 (-5.1; 12.1)	0.4
1–4 years	No JP	2000–2015	-5.9 (-11.6; 0.2)	0.1
5–9 years	No JP	2000–2015	-7.6 (-14.9; 0.4)	0.1
10–14 years	2004	2000–2004	7.6 (-27.7; 60.2)	0.7
		2004–2015	-8.5 (-15.9; -0.4)	< 0.0001
15–24 years	No JP	2000–2015	0.2 (-6.2; 7.2)	0.9
25–64 years	No JP	2000–2015	4.2 (-2.9: 11.8)	0.2
≥65 years	No JP	2000–2015	-4.5 (-12.3; 3.9)	0.3

*IMD* invasive meningococcal diseases, *APC* annual percent change, *CI* confidence interval, *JP* joinpoint

**Table 4:** Findings of the joinpoint regression for IPD by age group

Age group	Joinpoint	Years Range	APC (95% CI)	*p*-value
All age groups	No JP	2000–2015	0.7 (-0.6; 2.1)	0.3
<1 year	2004	2000–2004	15.5 (-25.5; 78.9)	0.5
		2004–2015	-16.7 (-28; -3.6)	< 0.0001
1–4 years	No JP	2000–2015	-7 (-13.7; 0.2)	0.1
5–9 years	No JP	2000–2015	0.8 (-9.5; 12.2)	0.9
10–14 years	2002	2000–2002	81.9 (79.6; 84.3)	< 0.0001
	2009	2002–2009	-20.4 (-20.6; -20.2)	< 0.0001
	2012	2009–2012	99 (96.4; 101.6)	< 0.0001
		2012–2015	-50.1 (-50.4; -49.7)	< 0.0001
15–24 years	2003	2000–2003	95.2 (-50.5; 670.3)	0.3
		2003–2015	-13.4 (-22.5; -3.3)	< 0.0001
25–64 years	No JP	2000–2015	0.8 (-1.8; 3.5)	0.5
≥65 years	No JP	2000–2015	1.7 (-0.5; 3.8)	0.1

*IPD* invasive pneumococcal diseases, *APC* annual percent change, *CI* confidence interval, *JP* joinpoint

**Table 5:** Findings of the joinpoint regression for invasive *H. influenzae* diseases by age group

Age group	Joinpoint	Years Range	APC (95% CI)	*p*-value
All age groups	No JP	2000–2015	-1.5 (-5.3; 2.4)	0.4
<1 year	2008	2000–2008	-17.5 (-30.5; -2.1)	< 0.0001
		2008–2015	10.8 (-12.9; 40.9)	0.4
1–4 years	2002	2000–2002	21.5 (-64.4; 314.2)	0.7
	2005	2002–2005	-44.4 (-92.5; 312.1)	0.5
		2005–2015	13.3 (0.0; 28.3)	< 0.0001
5–9 years	2008	2000–2008	7.6 (-1.3; 17.2)	0.1
		2008–2015	-15.1 (-24.0; -5.1)	< 0.0001
10–14 years	2003	2000–2003	17.2 (16.5; 18.0)	< 0.0001
	2009	2003–2009	-9.8 (-10.0; -9.5)	< 0.0001
	2012	2009–2012	23.4 (21.9; 24.9)	< 0.0001
		2012–2015	-20.6 (-21.1; -20.1)	< 0.0001
15–24 years	2003	2000–2003	-19.3 (-30.4; -6.5)	< 0.0001
		2003–2015	-0.1 (-2.1; 2.0)	0.9
25–64 years	No JP	2000–2015	-0.7 (-7.9; 7.1)	0.8
≥65 years	No JP	2000–2015	-1.4 (-7.1; 4.6)	0.6

*APC* annual percent change, *CI* confidence interval, *JP* joinpoint

### Health outcome analysis

The univariable analyses showed that there were more deaths in older patients, in females, in Italian patients, and in patients with comorbidities. Among all deaths due to IMD, only 9 (29%) were registered in the pediatric age as compared to 22 (71%) in people ≥18 years of age (*p* = 0.004). The same was observed for IPD (*p* < 0.0001) and invasive *H. influenzae* diseases (*p* = 0.003). Age was entered into all IBD logistic regression models and, in light of small absolute frequencies, it was classified as <18 years vs ≥18 years of age for IPD and as <65 years vs ≥65 years of age for invasive *H. influenzae* diseases. Gender was not shown to be significantly associated to death, but it was kept in the IMD and IPD logistic regression models. Among patients who died for IPD, there were more Italians than non-Italians (*p* = 0.052). Significant associations were not found for the other two IBD, therefore nationality was entered only into the logistic regression model for IPD. As for the Charlson Index, a smaller percentage of people without comorbidities was observed among patients who died as compared to those who survived. For instance, 90% of IMD patients discharged alive had no comorbidities in comparison to 74.2% among who died (*p* = 0.014). Significant associations were also seen in the other two IBD and Charlson Index was entered into all models as a dichotomous variable (presence/absence of comorbidities) (Table 6).

**Table 6:** Findings of univariable analyses performed by chi-square test

Variable	IMD			IPD			Invasive *H. influenzae* diseases		
	Dead n (%)	Alive n (%)	*p*-value	Dead n (%)	Alive n (%)	*p*-value	Dead n (%)	Alive n (%)	*p*-value
Age group									
<5 years	5 (16.1)	81 (27.1)	0.004	0 (0.0)	81 (8.4)	< 0.0001	1 (7.1)	15 (13.5)	0.003
5–17 years	4 (12.9)	63 (21.1)		1 (0.6)	30 (3.1)		0 (0.0)	7 (6.3)	
18–64 years	11 (35.5)	124 (41.5)		39 (24.1)	397 (41.1)		0 (0.0)	41 (36.9)	
≥65 years	11 (35.5)	31 (10.4)		122 (75.3)	459 (47.4)		13 (92.9)	48 (43.2)	
Gender									
male	10 (32.3)	145 (48.5)	0.085	77 (47.5)	514 (53.1)	0.185	7 (50.0)	61 (55.0)	0.726
female	21 (67.7)	154 (51.5)		85 (52.5)	453 (46.9)		7 (50.0)	50 (45.0)	
Nationality									
Italian	29 (96.7)	278 (93.6)	1.000	160 (98.8)	919 (95.4)	0.052	14 (100.0)	109 (98.2)	1.000
non-Italian	1 (3.3)	19 (6.4)		2 (1.2)	44 (4.6)		0 (0.0)	2 (1.8)	
Charlson Index									
0	23 (74.2)	269 (90.0)	0.014	88 (54.3)	643 (66.5)	0.004	6 (42.9)	64 (57.7)	0.090
1	7 (22.6)	19 (6.3)		41 (25.3)	153 (15.8)		1 (7.1)	22 (19.8)	
2	1 (3.2)	11 (6.7)		33 (20.4)	171 (17.7)		7 (50.0)	25 (22.5)	

*IMD* invasive meningococcal diseases, *IPD* invasive pneumococcal diseases

The final logistic regression models for type of invasive disease are shown in Table 7. They were overall statistically significant and demonstrated that older age was a risk factor for dying for all IBD. In particular, the IMD model was entirely explained by the variable age, with older age (≥65 years old) associated to a higher risk of dying (OR 3.13; 95% CI 1.14; 8.60) compared to adults (18–64 years old). As for the IPD model, adults and elderly (OR 17.43; 95% CI 2.40; 126.35) and patients with comorbidities (OR 1.45; 95% CI 1.03; 2.04) had a higher risk of death compared to patients in pediatric age and without comorbidities respectively. Similarly, to IMD, the model for invasive *H. influenzae* diseases was entirely explained by the variable age: elderly (≥65 years of age) showed a higher risk of death (OR 17.94; 95% CI 2.16; 148.71).

**Table 7:** Findings of multivariable logistic regression models

Type of IBD	Variable	OR (95% CI)	*p*-value
IMD (*N* = 330; *p* < 0.05; *R*^*2*^ = 0.07)	Age group		
	<5 years	0.76 (0.25; 2.30)	0.624
	5–17 years	0.79 (0.24; 2.61)	0.696
	18–64 years	1.00	
	≥65 years	3.13 (1.14; 8.60)	0.027
	Gender		
	male	0.70 (0.30; 1.62)	0.410
	female	1.00	
	Charlson Index		
	no comorbidities	1.00	
	comorbidities	1.58 (0.56; 4.42)	0.385
IPD (*N* = 1125; *p* < 0.001; *R*^*2*^ = 0.04)	Age group		
	<18 years	1.00	
	≥18 years	17.43 (2.40; 126.35)	0.005
	Gender		
	male	0.81 (0.58; 1.13)	0.217
	female	1.00	
	Nationality		
	Italian	3.38 (0.80; 14.20)	0.097
	non-Italian	1.00	
	Charlson Index		
	no comorbidities	1.00	
	comorbidities	1.45 (1.03; 2.04)	0.031
Invasive *H. influenzae* diseases (*N* = 125; *p* < 0.001; *R*^*2*^ = 0.16)	Age group		
	<65 years	1.00	
	≥65 years	17.94 (2.16; 148.71)	0.007
	Charlson Index		
	no comorbidities	1.00	
	comorbidities	0.87 (0.26; 2.92)	0.821

*IBD* invasive bacterial disease, *OR* odds ratio, *CI* confidence interval, *IMD* invasive meningococcal diseases, *IPD* invasive pneumococcal diseases

## Discussion

This historical observational study assessed the trends of IBD hospitalizations in a population of 3.7 million people over the past 16 years. The findings highlighted decreasing hospitalization rates for IPD in infants <1 year of age, likely because of the effects of PCV vaccines. A similar reduction in *S. pneumoniae*-related hospitalizations in children was shown in two other Italian regions, Friuli Venezia Giulia and Veneto [17]. Although data were limited, high PCV vaccination coverages retrieved for 2013–2015 support the conclusion. The data showed that, in the last few years, hospitalization rates for invasive *H. influenzae* diseases increased, as also reported by the National Surveillance System over the past 4 years. In fact, an increasing trend, although non-significant, was observed in infant <1 year of age from 2008 onwards, and a significantly increasing trend was shown in the 1–4 years age group from 2005 onwards. One can speculate that this increase was linked, among other reasons, to a drop in Hib vaccination coverage. Thanks to the high vaccination coverage reached in almost all Italian regions, cases attributable to serotype b, the only ones preventable by vaccination, are rare [26]. Nevertheless, the common belief that invasive *H. influenzae* diseases have disappeared after the introduction of the vaccine is not supported and should go no further. In fact, on average, one case out of nine per year (11.1%) occurred in children <4 years of age in 2012–2015. Overall, HRs for IPD were in line with national estimates, whereas HRs for invasive *H. influenzae* diseases, although in line with estimates reported by some Italian regions, were higher than national estimates presumably affected by underreporting [26]. A more complex situation emerged for IMD, whose HRs appeared to be, on average, higher than the national ones (0.6 per 100,000 vs 0.3 per 100,000) [26], with two high peaks in 2004–2005 and in 2015. Considering that the time-trend analysis also revealed joinpoints around those years, we can assume two crucial changes in the epidemiology of IMD in Tuscany. The first likely reflects the introduction of MCC vaccine in 2005 with ensuing reduction of hospitalizations especially in children from 1 to 4 years of age (although non-significant) and in adolescents from 10 to 14 years of age, both primary targets of the vaccination campaign. These findings were also in line with a recent time-trend analysis investigating the impact of the MCC vaccine introduction in Italy [39]. Nevertheless, not enough data on vaccination coverage were available and thus no relationship could be determined in changes in age-specific HRs in relation to vaccination. The second change is in line with the increasing number of cases reported in young adults by the National Surveillance System in 2015 and in the first quarter of 2016 [26]. This change was also seen in our data (in particular regarding the 15–24 years age group) with nine and three cases respectively compared to one case per year in the previous 2 years (data not shown). This brought to the implementation of extraordinary measures and to vaccinate, free of charge, people between 20 and 45 years of age and, under request and with co-payment, people above 45 years of age [28, 29]. It is important to note that, although no death occurred, 25 of the 330 IMD cases (7.6%) affected infants <1 year and that the trend of HR for this age group was the only positive one in the pediatric age (although non-significant). In Tuscany, as well as in other Italian regions, infants <1 years of age are not covered by the MCC vaccination that is offered at 13th months of age. The introduction of MenB vaccine for infants from 2014 onward can be expected to produce some benefits in years to come.

Regarding IBDs in adults, a note on IMD is warranted. Although the increased number observed in 2015–2016 has been mainly registered in young adults, adults from 25 to 64 years old contributed with half of the total cases per year: 16 cases in 2015 and six in the first quarter of 2016 as compared to three cases per year in the previous 2 years (data not shown). Time-trend analysis for this age group showed a positive but non-significant trend. Attention should be paid to this fast-changing situation also considering that adults above 45 years of age are not strictly a target of extraordinary MCC vaccination measures.

A deeper analysis is needed for the elderly. Despite the decreasing trend in IBD in vaccinated children (direct effect) and in unvaccinated subjects of all ages (indirect effect) [40–43], the level of disease control in the elderly is suboptimal. In fact, although no time-trends were observed, the absolute number of cases and CFRs remain high for all three IBD. For example, 42 of the 330 IMD cases (12.7%) occurred in people ≥65 years old, contributing with the highest number of deaths (11 out of 31 deaths due to IMD). This would appear even more relevant looking at invasive *H. influenzae* diseases: 61 out of the 125 cases (49%) were registered in elderly as well as all deaths except one. The age-specific CFRs were higher than European estimates reported by the European Centre for Disease Prevention and Control (ECDC) [1]: 26.2% vs 17.1% for IMD, 21% vs 14.3% for IPD, and 21.3% vs 15% for invasive *H. influenzae* diseases. Furthermore, in all three regression models, older age was significantly associated with a higher risk for death. Several studies in the literature found older age and Charlson comorbidities to be independent predictors of death [44, 45]. This evidence calls for actions to extend out high vaccination coverage in elderly and people with chronic conditions to prevent the occurrence of such IBD, in particular IPD, in these groups.

In fact, while MCC and Hib vaccines are not given to the elderly, the 23-valent pneumococcal polysaccharide vaccine (PPV23) first and then the PCV13 have been used in Italy in people ≥65 years of age. Nevertheless, albeit data on adults vaccination coverage are not routinely collected, local or regional studies suggest that vaccination coverage in people ≥65 years of age is quite low, varying from 0.7 to 50% between 2004 and 2008 [22], when the PPV23 was administered to elderly [46]. A recently concluded randomized trial in the Netherlands provide the missing evidence of PCV13 efficacy in preventing vaccine-type IPD in older adults [47]. This evidence, together with an increased awareness of the problem of IBD in the elderly, should support policy makers in their decisions on the implementation of pneumococcal vaccination. This is envisaged also because vaccinating elderly against *S. pneumoniae* may prevent not only IPD but also pneumonia, which causes 1 million hospitalizations in Europe, costs about €10 billion per year, and represents the most frequent cause of death from infection [48, 49].

A positive point of the present study is that it gives a thorough overview of the epidemiology of IBD yielding also CFRs. This measure is widely used as an outcome indicator to make comparisons over time and between areas as its calculation is less prone to bias [50]. Moreover, compared to the National Surveillance System which included invasive diseases since 2007 only [22], this study was able to provide a picture of IBD over a wider time window overcoming the apparent increase in the number of cases which occurred in the National Surveillance System. Our study presents also some limitations. One is concerning the general sparse-cells problem that makes joinpoint models unstable and may justify fluctuating positive and negative trends in children from 10 to 14 years of age both for IPD and invasive *H. influenzae* diseases. Furthermore, it should be kept in mind that in presence of zero-counts, small absolute fluctuation may have a great relative impact. Another limitation was the lack of information on death after discharge from the hospital or transfer to a private non-accredited institute even though we consider it improbable to affect our results as the number of transferred patients was very low. Another limitation is represented by the lack of information on serotype distribution and individual vaccination records that could have allowed a more in-depth analysis of the relationship between vaccination and the occurrence of diseases. Additionally, over the past 16 years, diagnostics methods have become more sensitive and life support techniques could have influenced the health outcome. Finally, R-square values of regression models were very low, ranging between 4 and 16% because of the limited number of variables available from the hospital discharge records.

In conclusion, the results of our study contribute to the body of evidence on the epidemiology of IBD and the importance of ensuring high vaccination coverage. A constant effort should be made to attain and maintain high vaccination coverage among children in order to further reduce the incidence of all IBD and control apparent increasing trends. In particular, attention should be paid to the increase in invasive *H. influenzae* diseases and to the changing epidemiological scenario of IMD. Furthermore, actions should be also promoted to implement vaccination in the elderly. Eventually, prevention remains the most valuable tool to help reducing the burden of IBD in all age groups.

## Conclusion

This study shows changes in the epidemiology of IBD, particularly due to *H. influenzae* and *N. meningitidis,* and high rates of hospitalizations and deaths for all types of IBD in elderly. This evidence calls for actions in order to maintain high vaccination coverage among children and promote vaccination in older age groups.

## Additional files

Additional file 1: **Table S1.** Characteristics of patients with IBD resident in Tuscany in 2000–2016 (*N* = 1584). (DOCX 12 kb)

Additional file 2: **Figure S1.** Joinpoint regression of IMD HRs, all ages, years 2000–2015. (TIF 61 kb)

## Abbreviations

4CMenB: multicomponent MenB vaccine
APC: Annual Percentage Change
CFR: case-fatality rates
CI: confidence interval
ECDC: European Centre for Disease Prevention and Control
Hib: H. influenzae serotype b
HR: hospitalization rates
IBD: invasive bacterial diseases
ICD-9-CM: International Classification of Disease, Ninth Revision, Clinical Modification
IMD: invasive meningococcal diseases
IPD: invasive pneumococcal diseases
JP: joinpoint
MCC: MenC conjugate vaccine
MenB: meningococcal serogroup B
MenC: meningococcal serogroups C
MenY: meningococcal serogroup Y
OR: Odds Ratio
PCV13: 13-valent pneumococcal conjugate vaccine
PCV7: 7-valent pneumococcal conjugate vaccine
SE: standard error
