# Supplementary material for: Hospitalization rates and outcome of invasive bacterial vaccine-preventable diseases in Tuscany: a historical cohort study of the 2000–2016 period
Source: BMC Infect Dis. 2018 Aug 13;18:396. doi: 10.1186/s12879-018-3316-1 (PMC6090664; doi:10.1186/s12879-018-3316-1)
Supplement: Supplementary file 1 — Table S1. Characteristics of patients with IBD resident in Tuscany in 2000–2016 (N = 1584). (DOCX 12 kb) [file 12879_2018_3316_MOESM1_ESM.docx]

| Characteristic | Children and adolescents (<18) (n=288) | Adults and elderly (≥18) (n=1,296) |
| --- | --- | --- |
| Type of IBD− n (%) |  |  |
| IMD | 153 (53.1) | 177 (13.6) |
| IPD | 112 (38.9) | 1,017 (78.5) |
| Invasive *H. influenzae* disease | 23 (8.0) | 102 (7.9) |
| Gender − n (%) |  |  |
| male | 164 (56.9) | 650 (50.1) |
| female | 124 (43.1) | 646 (49.9) |
| Age − mean ±SD | 4.8 ± 5.2 | 61.3 ± 18.9 |
| Length of hospitalization − mean ±SD | 16.7 ± 15.4 | 12.1 ± 8.9 |
| Nationality − n (%) |  |  |
| Italian | 265 (92.0) | 1,244 (96.0) |
| Non-Italian | 19 (6.6) | 49 (3.8) |
| European (excl Italians) | 10 (3.5) | 19 (1.5) |
| African | 7 (2.4) | 13 (1.0) |
| Asian | 1 (0.4) | 7 (0.5) |
| North American | 0 (0) | 0 (0) |
| South and Central American | 1 (0.4) | 10 (0.8) |
| Missing | 4 (1.4) | 3 (0.2) |

IBD: invasive bacterial disease; IMD: invasive meningococcal disease; IPD: invasive pneumococcal disease; SD: standard deviation.
