# Supplementary figures and images for: Hospitalization rates and outcome of invasive bacterial vaccine-preventable diseases in Tuscany: a historical cohort study of the 2000–2016 period
Source: BMC Infect Dis. 2018 Aug 13;18:396. doi: 10.1186/s12879-018-3316-1 (PMC6090664; doi:10.1186/s12879-018-3316-1)

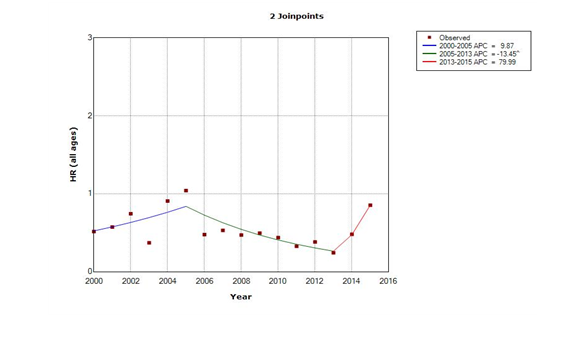

Supplement: Supplementary file 2 — Figure S1. Joinpoint regression of IMD HRs, all ages, years 2000–2015. (TIF 61 kb) [file 12879_2018_3316_MOESM2_ESM.tif]
